# Combined Treatment with Bojungikgi-Tang and Riluzole Regulates Muscle Metabolism and Dysfunction in the hSOD1^G93A^ Mouse Model

**DOI:** 10.3390/antiox11030579

**Published:** 2022-03-18

**Authors:** Mudan Cai, Eun Jin Yang

**Affiliations:** KM Science Research Division, Korea Institute of Oriental Medicine (KIOM), Yuseong-daero 1672, Yuseong-gu, Daejeon 34054, Korea; mudan126@kiom.re.kr

**Keywords:** amyotrophic lateral sclerosis (ALS), Bojungikgi-tang, riluzole, gastrocnemius, spinal cord, metabolism, inflammation

## Abstract

The progressive neurodegenerative disease, amyotrophic lateral sclerosis (ALS), is characterized by muscle weakness and atrophy owing to selective motoneuron degeneration. The anti-glutamatergic drug, riluzole (RZ), is the standard-of-care treatment for ALS. Bojungikgi-tang (BJIGT), a traditional herbal formula, improves motor function and prolongs the survival of mice with ALS. As ALS is a multicomplex disease, effective therapies must target multiple mechanisms. Here, we evaluated the efficacy of a BJIGT/RZ combination (5-week treatment) in 2-month-old hSOD1^G93A^ mice with ALS. We performed quantitative polymerase chain reaction, Western blotting, immunohistochemistry, and enzyme activity assays. BJIGT/RZ significantly attenuated inflammation, autophagy, and metabolic and mitochondrial dysfunctions in the gastrocnemius (GC) compared with the control. It reduced the mRNA and protein levels of muscle denervation-related proteins and creatine kinase levels. The total creatine level was significantly higher in the BJIGT/RZ-treated GC. Moreover, after BJIGT/RZ treatment, the number of Nissl-stained motoneurons and choline acetyl transferase-positive neurons in the spinal cord significantly increased via the regulation of proinflammatory cytokines. Collectively, the BJIGT/RZ treatment was superior to single-drug treatments in alleviating multiple ALS-related pathological mechanisms in the ALS mouse model. Overall, BJIGT can serve as a dietary supplement and be combined with RZ to achieve superior therapeutic effects against ALS.

## 1. Introduction

Amyotrophic lateral sclerosis (ALS) is a progressive fatal neurodegenerative disease characterized by muscle weakness and atrophy in both upper and lower motor neurons owing to motor neuron degeneration. The incidence of ALS is 2 cases per 100,000 person-years, and patients with ALS die because of respiratory failure within 2–4 years of diagnosis [1]. Several studies have shown that glutamate is elevated in the plasma, serum, cerebrospinal fluid (CSF), and postmortem brain tissue of patients with ALS [2]. In ALS, increased glutamate-mediated excitotoxicity, neuroinflammation, mitochondrial dysfunction, oxidative stress, and metabolic dysfunction lead to the death of motor neurons and muscle degeneration [3]. Moreover, activated astrocytes and microglia release proinflammatory cytokines and toxic factors, contributing to neurotoxicity [4]. Dysregulation of energy metabolism by oxidative damage plays a key role in the onset and progression of ALS [5]. Thus, multiple mechanisms have been identified as potential therapeutic targets for ALS in preclinical studies, but drug compounds that target a single mechanism have mostly failed in clinical trials [6]. These failures have led to an unmet need for effective treatments for ALS.

Riluzole (RZ) is a neuroprotective drug that inhibits glutamatergic neurotransmission in the central nervous system (CNS) [7]. RZ also inhibits the release of glutamate from presynaptic terminals and blocks the postsynaptic N-methyl-D-aspartate (NMDA) receptors [8]. RZ was first approved by the United States (US) Food and Drug Administration (FDA) in 1995; it is commonly prescribed for patients with ALS. RZ treatment protects motor function and prolongs survival in mice with familial ALS [9]. Treatment with RZ can extend the lifespan by 2–3 months, but it is not effective in arresting disease progression in patients with ALS [10]. However, a recent clinical study showed that RZ prolongs the survival of patients in the last stage of disease, but further research is required to confirm the treatment effects [11]. The abovementioned findings indicate that RZ provides only modest benefits and that the benefits are observed only in some patients. Considering that multiple pathogenic mechanisms likely underlie the progression of ALS, the use of RZ in combination with other pharmacological agents may increase the therapeutic efficacy of RZ.

Herbal medicine has been widely used in Asia for over 2000 years. The pharmacological effects of numerous herbal medicines have been demonstrated in numerous studies using a variety of techniques. Bojungikgi-tang (BJIGT; Bu Zhong Yi Qi Tang in China; Hochuekkito in Japan) is a well-known traditional herbal formula that is commonly used to reduce fatigue and enhance immune function. The essential herbal components of BJIGT are Angelicae Gigantis radix, Astragali radix, Atractylodis rhizome, Bupleuri radix, Cimicifugae rhizome, Citri unshii pericarpium, Ginseng radix alba, and Glycyrrhizae radix. Astragalus polysaccharides and saponins (e.g., astragaloside IV) are the major active compounds of Astragali Radix, which can regulate immune and respiratory diseases, and exert neuroprotective effect on the CNS [12]. The polysaccharides in Ginseng radix also regulate immune functions, tumor, and oxidative stress; they exhibit antifatigue and antidepression effects [13]. Lim et al. reported that glycyrrhizin is the compound with the most amount among the seven standard components (liquiritin apioside, liquiritin, nodakenin, hesperidin, glycyrrhizin, decursin, and decursinol angelate) in BJIGT [14]. In addition, BJIGT reportedly ameliorates immune system dysfunction, fatigue, aging, gastric injury, respiratory diseases, and cognitive impairment [14]. Previously, we reported that BJIGT improves motor function and prolongs survival through anti-inflammatory and antioxidative stress activities, and via the regulation of autophagy dysfunction in hSOD1^G93A^ mice [15]. These findings suggest that BJIGT has multitarget effects and can be applied to diseases with multiple underlying mechanisms. Wang et al. [16] and Sun and Xu [17] demonstrated that Buzhongyiqitang jiajian combined with other treatments improves muscle strength and appetite, and reduces fatigue in patients with ALS. Therefore, considering that multiple mechanisms underlie ALS pathology, combination therapies that address multiple targets hold promise for achieving maximal therapeutic effects. In this study, we show that RZ combined with BJIGT exerts neuroprotective effects, promotes anti-inflammatory activity, and regulates the dysfunction of energy metabolism in an ALS animal model.

## 2. Materials and Methods

### 2.1. Animals

The hemizygous hSOD1^G93A^ transgenic (Tg) mouse model with a single amino acid substitution of glycine with alanine at codon 93 of the human *SOD1* gene is commonly used as an ALS animal model [18]. Male hSOD1^G93A^ Tg mice were purchased from Jackson Laboratory (Bar Harbor, ME, USA). Three mice were housed in specific pathogen-free control conditions at a constant temperature (21 ± 3 °C) and humidity (50 ± 10%) with a 12 h light/dark cycle (lights on 07:00–19:00 h and lights off 19:00–07:00 h). Water and food were provided ad libitum. The care and treatment of animals were per the animal care guidelines of the Korea Institute of Oriental Medicine (protocol number: 20–204). In this study, 2-month-old hSOD1^G93A^ Tg mice were randomly grouped into four groups: a distilled water (DW)-administered group (control), a BJIGT-treated group (BJIGT), an RZ-treated group (RZ), and a combined BJIGT/RZ-treated group (BJIGT/RZ).

### 2.2. BJIGT and RZ Treatment

BJIGT and RZ were purchased from Hankookshinyak (Chungnam, Korea) and Calbiochem (Darmstadt, Germany), respectively. BJIGT and RZ were prepared as reported previously [19]. BJIGT (1 g/kg) was orally administered once daily for 5 weeks to mice in the BJIGT group. RZ (8 μg/g) was administered orally every other day for 5 weeks to mice in the RZ group. The mice in the BJIGT/RZ group were simultaneously administered RZ and BJIGT, according to the aforementioned protocols for 5 weeks.

### 2.3. Tissue Preparation

After 5 weeks of drug treatment, the mice were deeply anesthetized using avertin (Sigma, St. Louis, MO, USA) and transcardially perfused with phosphate-buffered saline (PBS). The gastrocnemius (GC) and spinal cord (SP) tissues were collected. In addition, the SP was fixed in 4% paraformaldehyde at 4 °C for 4 days, and embedded in paraffin. The paraffin blocks were transversely cut into 10 µm-thick sections for staining. 

### 2.4. Western Blotting

Western blotting was performed as described previously [15]. The GC and SP tissues were homogenized in radioimmunoprecipitation assay buffer (Biosesang, Seongnam, Korea) containing phosphatase and proteinase inhibitors (Thermo Fisher Scientific, Waltham, MA, USA). The protein samples (20 µg protein) were separated on Bolt 4–12% Bis-Tris Plus gels (Thermo Fisher Scientific, Waltham, MA, USA) and transferred to polyvinylidene difluoride membranes (Bio-Rad Laboratories, Hercules, CA, USA). The membranes were incubated with primary and matched secondary antibodies to identify the indicated proteins. Finally, the protein bands were visualized using a ChemiDoc imaging system (Bio-Rad Laboratories) with an enhanced chemiluminescence kit (Thermo Fisher Scientific, Waltham, MA, USA) and quantified using ImageJ (version 1.46j; National Institutes of Health [NIH], Bethesda, MD, USA). For Western blotting, the following primary antibodies were used: cluster of differentiation 11b (CD11b), interleukin 1 beta (IL-1β), interleukin 6 (IL-6), ATG7, mitofusin (MFN), dynamin-related protein 1 (DRP1), glucose transporter type 4 (GLUT4), prospero homeobox protein 1 (Prox1), and α-tubulin (all from Abcam, Cambridge, MA, USA; diluted 1:1000); microtubule-associated protein 1A/1B light chain (LC)3B, BCL-2–associated athanogene 3 (BAG3), AKT, phosphorylated AKT (pAKT), mechanistic target of rapamycin (MTOR), phosphorylated MTOR (pMTOR), and transforming growth factor-β (TGF-β) (all from Cell Signaling Technology, Danvers, MA, USA; diluted 1:1000); peroxisome proliferator-activated receptor gamma coactivator 1-alpha (PGC1-α) (all from Thermo Fisher Scientific, Waltham, MA, USA; diluted 1:1000); and glyceraldehyde-3-phosphate dehydrogenase (GAPDH) (Santa Cruz Biotechnology, Santa Cruz, CA, USA; diluted 1:1000).

### 2.5. Nissl Staining and Immunohistochemistry

For staining, the SP tissue sections were deparaffinized in xylene, rehydrated in a decreasing alcohol gradient (100%, 95%, and 70% alcohol), and finally in water (5 min each, repeated twice), and then washed again in PBS. Nissl staining was performed as described previously [15]. Following deparaffinization, the sections were stained with 0.1% cresyl violet (Sigma, St. Louis, MO, USA) for 5 min. Subsequently, the sections were covered with a coverslip using Histomount medium. To quantify Nissl staining, images of the ventral horn of the SP were captured, and Nissl-stained motor neurons were counted in a blinded manner using Image J (version 1.46j) as described previously [15].

Immunohistochemical staining was performed as previously described [15]. After deparaffinization, the SP sections were incubated with 3% H_2_O_2_, and then blocked with 5% bovine serum albumin in 0.01% PBS-Triton X-100. The slides were then incubated with primary antibodies against choline acetyl transferase (ChAT) (diluted 1:5000; AbcamCambridge, MA, USA), ionized calcium-binding adaptor molecule 1 (Iba-1) (diluted 1:1000; Wako, Osaka, Japan), and glial fibrillary acid protein (GFAP) (diluted 1:1000; Agilent Technologies, Santa Clara, CA, USA) overnight at 4 °C. The next day, the slides were incubated with primary matched-secondary antibodies. For visualization, the slides were treated with ABC solution (Vector Laboratories, Burlingame, CA, USA) and visualized using 3,3′-diaminobenzidine peroxidase substrate solution (Vector Laboratories). Coverslips were mounted onto the SP slides using Vectashield mounting medium (Vector Laboratories) and images were captured using a light microscope (Olympus BX51, Tokyo, Japan). Cell counts are expressed as ChAT-positive motor neurons in the ventral horn of the L4–5 region of the SP. For quantification, the intensities of Iba-1- and GFAP-positive cells were determined using ImageJ (version 1.46j; NIH). 

### 2.6. RNA Extraction and Real-Time Reverse Transcription Polymerase Chain Reaction

For RNA extraction, the GC and SP tissues were homogenized in lysis buffer provided as a part of a total RNA extraction kit (Intron Biotechnology, Seongnam-Si, Korea); RNA extraction was performed according to the manufacturer’s protocol. The RNA concentration in the samples was measured using spectrometry (NanoDrop One; Thermo Fischer Scientific, Wilmington, DE, USA). For cDNA synthesis, 1 µg of RNA from each sample was reverse transcribed to cDNA using an iScript cDNA synthesis kit (Bio-Rad Laboratories), according to the manufacturer’s instructions. After cDNA synthesis, each sample template was added to a mixture of SYBR supermix (Bio-Rad Laboratories), primers, and nuclease-free H_2_O. The RT-PCR was performed with the following cycle parameters: 95 °C for 30 s, 40 cycles at 95 °C for 15 s, and 60 °C for 1 min. The primers were synthesized by Bioneer Corp. (Daejeon, Republic of Korea); the primers used in this study were as follows: *Phkg2* (Accession No: NM_026888); *Pygm* (Accession No: NM_011224); *Aco1* (Accession No: NM_007386); *Aco2* (Accession No: NM_080633); *MuSk* (Accession No: NM_001037127); *Chrng* (Accession No: NM_009604); *Myog* (Accession No: NM_031189); *IL-1β* (Accession No: NM_008361); *TNF-α* (Accession No: XM_021218152); *IL-6* (Accession No: NM_031168); *IFN-γ* (Accession No: NM_008337); *GAPDH* (Accession No: XM_036165840). *GAPDH* was used as the housekeeping gene.

### 2.7. ATP Level, Creatine Level, and Creatine Kinase Assays

For the ATP level, creatine level, and creatine kinase (CK) assays, the GC tissues were homogenized with the assay buffers provided with an ATP colorimetric assay kit (BioVision, Milpitas, CA, USA), a creatine assay kit (BioVision, Milpitas, CA, USA), and a CK assay kit (Bioassay Systems, Hayward, CA, USA), respectively. The assays were performed according to the manufacturers’ protocols. Plates containing standards and samples were read in a plate reader (Molecular Devices, San Jose, CA, USA) at the proper absorption wavelength (570 nm or 340 nm) for each assay. The total protein concentration was determined using a bicinchoninic acid protein assay kit (Thermo Fisher Scientific, Waltham, MA, USA).

### 2.8. Statistical Analysis

Data are presented as mean ± standard error of the mean (SEM). The results were analyzed using the one-way analysis of variance followed by Tukey’s test for multiple comparisons using Prism v.9.0 (GraphPad, La Jolla, CA, USA).

## 3. Results

### 3.1. Combined BJIGT/RZ Treatment Affects Inflammation and Autophagy Dysfunction in the GC of the hSOD1^G93A^ ALS Mouse Model

**Figure 1:**
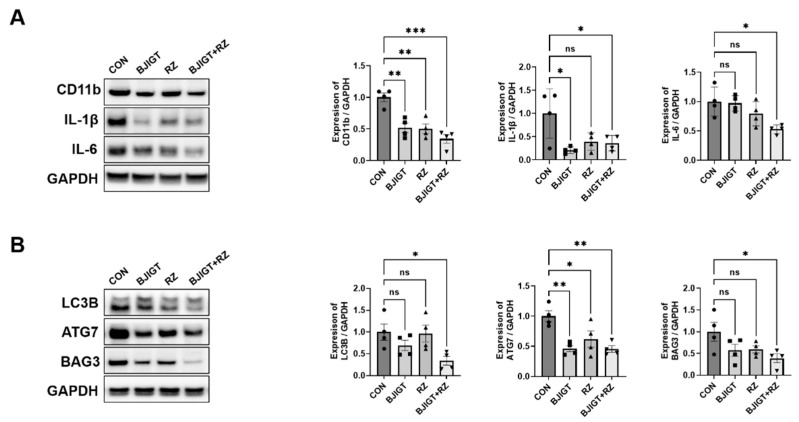
Combined BJIGT/RZ treatment regulates inflammation and autophagy dysfunction in the GC of hSOD1^G93A^ amyotrophic lateral sclerosis (ALS) mice. (**A**) Representative Western blots depicting alterations in inflammation-related proteins including cluster of differentiation 11b (CD11b), interleukin 1β (IL-1β), and IL-6 in the GC of control, BJIGT, RZ, and combined BJIGT/RZ treatment groups of hSOD1^G93A^ mice. Glyceraldehyde-3-phosphate dehydrogenase (GAPDH) was used as a loading control to normalize protein levels. Quantification of bands showing the levels of CD11b, IL-1β, and IL-6 in the drug treatment group compared with that in the control group (*n* = 4/group). (**B**) Representative Western blots showing the alterations in autophagy-related proteins including microtubule-associated protein 1A/1B light chain 3B (LC3B), autophagy related 7 (ATG7), and BCL-2–associated athanogene 3 (BAG3) in the GC of each group of hSOD1^G93A^ mice. GAPDH was used as a loading control to normalize the protein levels. Quantification of bands pertaining to LC3B, ATG7, and BAG3 in the drug treatment groups compared with those in the control group (*n* = 4/group). Data are normalized to the control groups and presented as mean ± SEM. ns: not significant, * *p* < 0.05, ** *p* < 0.01, and *** *p* < 0.001, compared with the control group. Distilled water (DW)-administered transgenic (Tg) mice (CON), BJIGT-treated Tg mice (BJIGT), RZ-treated of Tg mice (RZ), and combined BJIGT/RZ-treated Tg mice (BJIGT+RZ). The different number of circles, squares and triangles are indicated the number of sample sot.

To examine the effect of combined BJIGT/RZ treatment on the hSOD1^G93A^ ALS mouse model, BJIGT, RZ, and BJIGT/RZ were administered for 5 weeks to 2-month-old Tg mice. First, to determine whether the combination treatment was more effective in modulating the inflammation pathway, we measured inflammation-related proteins and proinflammatory cytokines in the GC of hSOD1^G93A^ mice with ALS. The expression of the microglial cell marker (CD11b) was significantly reduced to 48%, 50%, and 66% in the BJIGT, RZ, and combination treatment groups, respectively, compared with that in the control group. Additionally, compared with that in the control group, the expression of proinflammatory cytokines including IL-1β and IL-6 was significantly decreased by 64% and 47%, respectively, in the combination treatment group (Figure 1A). However, as shown in Figure 1A, in the single treatment groups (BJIGT or RZ), the IL-6 level in the GC tissue did not decrease considerably compared with that in the control group.

Crippa et al. demonstrated that the autophagy pathway was activated in the skeletal muscle of SOD1^G93A^ ALS mice in the late disease stage. The autophagic response induced by the *SOD1* mutation is considerably higher in the muscle than in the SP [20]. To evaluate the effect of the combination treatment on the autophagy pathway, we examined the levels of autophagy-related proteins. The levels of LC3B, ATG7, and BAG3 (autophagy-related proteins) in the GC were significantly decreased by 66%, 55%, and 61%, respectively, in the combination treatment group compared with those in the control group (Figure 1B). These results suggest that the combined BJIGT/RZ treatment inhibits the inflammatory response by regulating autophagy dysfunction.

### 3.2. Combined BJIGT/RZ Treatment Ameliorates Metabolic Dysfunction in the GC of the hSOD1^G93A^ ALS Mouse Model

**Figure 2:**
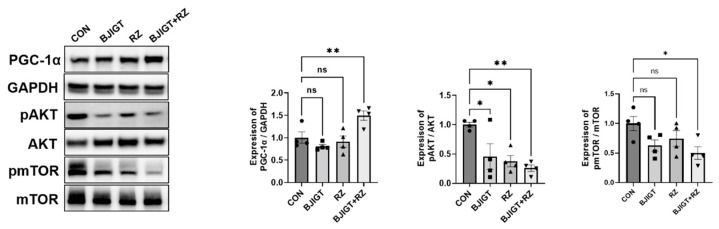
Combined BJIGT/RZ treatment ameliorates metabolism dysfunction in the GC of the hSOD1^G93A^ ALS mouse model. Representative Western blots depicting the alteration in metabolism-related proteins including peroxisome proliferator-activated receptor gamma coactivator 1-alpha (PGC-1α), pAKT, and phosphorylated mammalian target of rapamycin (pmTOR) in the GC of each group of hSOD1^G93A^ mice. Glyceraldehyde-3-phosphate dehydrogenase (GAPDH) was used as a loading control to normalize the protein levels. Quantification of immune blots; the level of PGC-1α was normalized to that of GAPDH and the levels of pAKT and pmTOR were normalized to the total AKT and total mTOR levels, respectively; the drug treatment groups were compared with the control group (*n* = 4/group). Data are normalized to the control group data and presented as mean ± SEM. ns: not significant, * *p* < 0.05, ** *p* < 0.01, compared with the control group. Distilled water (DW)-administered transgenic (Tg) mice (CON), BJIGT-treated Tg mice (BJIGT), RZ-treated Tg mice (RZ), and combined BJIGT/ RZ treated Tg mice (BJIGT+RZ). The different number of circles, squares, and triangles are indicated the number of sample sot.

Metabolic dysregulation is a part of ALS pathology [21]. Therefore, we examined the effect of the combination treatment on the metabolic pathways in the GC of hSOD1^G93A^ ALS mice. As shown in Figure 2, the level of PGC-1α in the GC were considerably increased (by 50%) in the combination treatment group compared with that in the control group. In addition, the combination treatment significantly reduced the expression of pAKT and pmTOR by 74% and 50% in the GC, respectively, compared with that in the control group. These findings indicate that the combined treatment regulates metabolic dysfunction in ALS mice. Such a metabolic regulation may affect energy metabolism via the regulation of mitochondrial dysfunction.

### 3.3. Combined BJIGT/RZ Treatment Regulates Mitochondrial Dysfunction in the GC of hSOD1^G93A^ ALS Mouse Model

**Figure 3:**
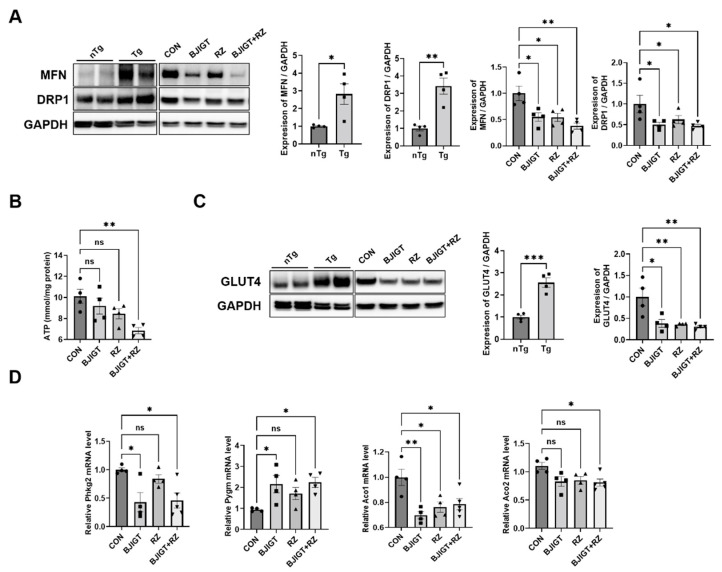
Combined BJIGT/RZ treatment ameliorates mitochondrial dysfunction in the GC of the hSOD1^G93A^ALS mouse model. (**A**) Representative Western blots depicting the alteration in mitochondrial dynamic-related proteins including mitofusin (MFN) and dynamin-related protein (DRP1), in the GC of the control, BJIGT, RZ, and combined BJIGT/RZ treatment groups of hSOD1^G93A^ mice. GAPDH was used as a loading control to normalize the protein levels. Quantification of bands showing the levels of MFN and DRP1 in the drug treatment groups compared with those in the control group (*n* = 4/group). (**B**) Quantitative analysis of the ATP levels in the GC of each group of hSOD1^G93A^ mice (*n* = 4/group). (**C**) Representative Western blots and quantification of the bands pertaining to glucose transporter type 4 (GLUT4) in the GC of each group of hSOD1^G93A^ mice. Glyceraldehyde-3-phosphate dehydrogenase (GAPDH) was used as the loading control. (**D**) Quantitative analysis of the relative mRNA expression of enzymes involved in glycogenolysis and tricarboxylic acid (TCA) cycle, including phosphorylase kinase (*Phkg2*), glycogen phosphorylase (*Pygm*), and aconitases (*Aco1 and Aco2*), in the GC of each group of hSOD1^G93A^ mice (*n* = 4–5/group). Data are normalized to the control group data and presented as mean ± SEM. ns: not significant, * *p* < 0.05, ** *p* < 0.01, and *** *p* < 0.001, compared with the control group, DW-administered transgenic (Tg) mice (CON), BJIGT-treated Tg mice (BJIGT), RZ-treated Tg mice (RZ), and combined BJIGT/ RZ-treated Tg mice (BJIGT+RZ). The different number of circles, squares, and triangles are indicated the number of sample sot.

Mitochondrial dysfunction is well known in the skeletal muscles of patients with ALS and in mice with ALS [22]. Luo et al. [23] reported that mitochondrial dynamics were defective in the skeletal muscle of SOD1^G93A^ mice with ALS. Therefore, we first examined the alterations in mitochondrial dynamic-related proteins (MFN and DRP1) between the nTg and Tg groups. Our results showed that the levels of MFN and DRP1 in the GC were significantly increased by 180% and 240% in the Tg group compared with those in the age-matched nTg group. Moreover, the combination treatment significantly led to a decrease in the expression of MFN and DRP1 by 62% and 53%, respectively, compared with that in the control group (Figure 3A). These results suggest that the combination treatment modulates mitochondrial dysfunction in the GC of ALS mice. Next, we determined whether the combination treatment affected the ATP level in the GC. As shown in Figure 3B, the combination treatment significantly decreased the ATP level by 32% compared with that in the control group. In addition, we observed that the level of GLUT4 (muscle-related glucose transporter) in the GC was significantly increased (by 150%) in the Tg group compared with that in the age-matched nTg group. Moreover, the expression of GLUT4 was reduced by 62%, 64%, and 69% in the BJIGT, RZ, and combination treatment groups, respectively, compared with that in the control group (Figure 3C). We also evaluated the mRNA expression of glycogenolysis and glycolysis regulatory enzymes in the GC. Glycogen phosphorylase (*Pygm*) is a key enzyme involved in the first step of glycogenolysis. As shown in Figure 3D, the level of the glycogenolysis regulatory enzyme phosphorylase kinase (*Phkg2*) was significantly decreased by 54% in the combination treatment group compared with that in the control group. The level of *Pygm* was increased by 57% and that of the tricarboxylic acid (TCA) cycle metabolism aconitase enzymes (Aco1 and Aco2) was significantly decreased by 21% and 19%, respectively, in the combination treatment group compared with those in the control group. These results suggest that the combined BJIGT/RZ treatment alleviates mitochondrial dysfunction by regulating the dynamics and ATP synthesis in the GC of the hSOD1^G93A^ mice with ALS.

### 3.4. Combined BJIGT/RZ Treatment Attenuates Muscle Denervation in the GC of the hSOD1^G93A^ ALS Mouse Model

**Figure 4:**
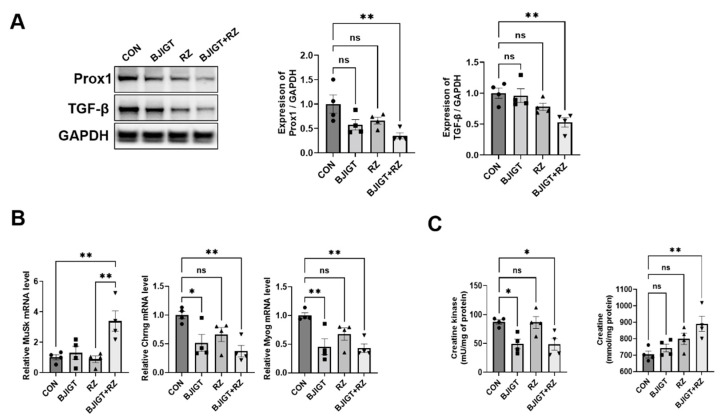
Combined BJIGT/RZ treatment improves muscle denervation in the GC of the hSOD1^G93A^ ALS mouse model. (**A**) Representative Western blots depicting the alteration of muscle denervation-related proteins, including prospero homeobox protein 1 (Prox1) and transforming growth factor-β (TGF-β), in the GC of each group of hSOD1^G93A^ mice. GAPDH was used as the loading control to normalize the protein levels. Quantification of bands showing the levels of Prox1 and TGF-β in the drug treatment groups compared with those in the control group (*n* = 4/group). (**B**) Quantitative analysis of the relative mRNA expression of genes involved in muscle denervation including muscle-specific kinase (*MuSK*), cholinergic receptor nicotinic gamma subunit (*Chrng*), and myogenin (*Myog*) in the GC of each group of hSOD1^G93A^ mice (*n* = 4/group). (**C**) Quantitative analysis of the levels of CK and creatine in the GC of each group of hSOD1^G93A^ mice (*n* = 4/group). Data are normalized to the control group data and presented as mean ± SEM. ns: not significant, * *p* < 0.05 and ** *p* < 0.01, compared with the control group. DW-administered transgenic (Tg) mice (CON), BJIGT-treated Tg mice (BJIGT), RZ-treated Tg mice (RZ), and combined BJIGT/ RZ-treated Tg mice (BJIGT+RZ). The different number of circles, squares, and triangles are indicated the number of sample sot.

As ALS pathological mechanisms, including inflammation, autophagy dysfunction, and mitochondrial dysfunction, initiate muscle denervation and death of motor neurons, we investigated whether the combined BJIGT/RZ treatment modulates muscle denervation in the GC of hSOD1^G93A^ ALS mice. As shown in Figure 4A, the expression of Prox1 and TGF-β was considerably decreased by 65% and 47%, respectively, in the combination treatment group compared with that in the control group. In addition, the combined BJIGT/RZ treatment significantly increased the mRNA level of muscle-specific kinase (*MuSK*), which protects against denervation in the GC by 240% compared with the level in the control group, and by 290% compared with the level in the RZ group (Figure 4B). The combination treatment also led to a significant decrease in the mRNA levels of cholinergic receptor nicotinic gamma subunit (*Chrng*) and myogenin (*Myog*) (which increase during denervation) in the GC by 63% and 57%, respectively, compared with the levels in the control group (Figure 4B). The level of CK reportedly increases in patients with ALS; this increase is associated with motor neuron loss, denervation, and muscular atrophy. As shown in Figure 4C, the combination treatment significantly reduced the activity of CK in the GC by 45% compared with that in the control group. Furthermore, the level of creatine was also significantly increased by 27% in the GC in the combination treatment group compared with that in the control group. Collectively, these findings show that the combined BJIGT/RZ treatment protects against muscle denervation by increasing the GC creatine level in hSOD1^G93A^ ALS mice.

### 3.5. Combined BJIGT/RZ Treatment Protects Motor Neurons and Reduces Neuroinflammation in the SP of the hSOD1^G93A^ ALS Mouse Model

Next, we evaluated whether the combined BJIGT/RZ treatment has neuroprotective effects in the SP in hSOD1^G93A^ ALS mice.

**Figure 5:**
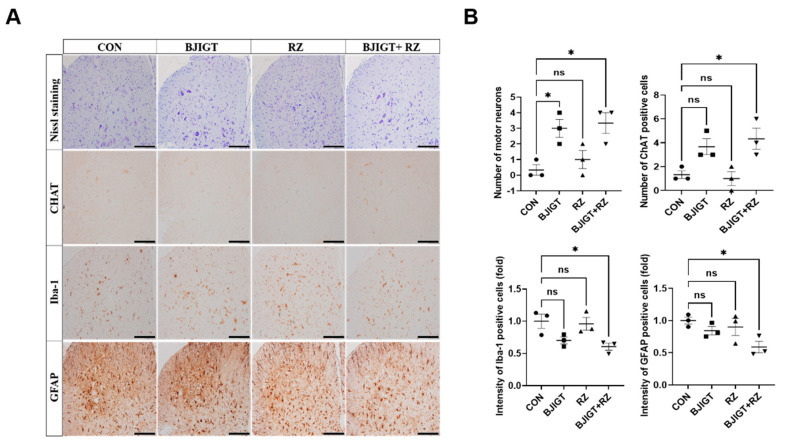

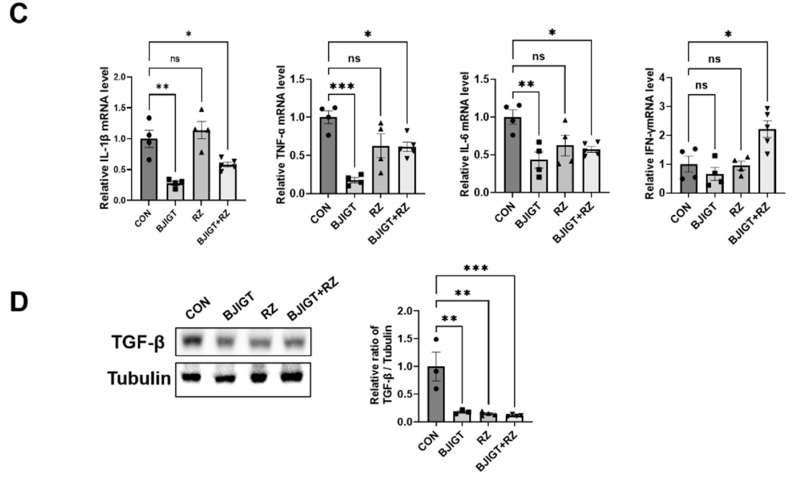
Combined BJIGT/RZ treatment protects motor neurons and reduces neuroinflammation in the spinal cord (SP) of the hSOD1^G93A^ ALS mouse model. (**A**) Photomicroscopic images of Nissl staining and immunostaining of choline acetyl transferase (ChAT), ionized calcium-binding adaptor molecule 1 (Iba-1), and glial fibrillary acid protein (GFAP) in the SP of each group of hSOD1^G93A^ ALS mice. (**B**) Quantification of the number of Nissl-stained motor neurons and ChAT-positive neurons (maker for cholinergic neuron), and the intensity of Iba-1 positive cells (marker for microglial cells) and GFAP-positive cells (marker for astrocytes) (*n* = 4–5/group) (scale bar, original magnification ×200; bar represents 200 μm). (**C**) Quantitative analysis of the relative mRNA expression of the proinflammatory cytokines interleukin 1β (*IL-1β*), tumor necrosis factor α (*TNF-α*), *IL-6*, and interferon γ (*IFN-γ*) in the GC of each group of hSOD1^G93A^ mice (*n* = 4–5/group). (**D**) Representative immunoblots and quantification of the bands pertaining to transforming growth factor β (TGF-β) in the GC of each group of hSOD1^G93A^ mice. Tubulin was used as the loading control. Data are normalized to the control group data and presented as mean ± SEM. ns: not significant, * *p* < 0.05, ** *p* < 0.01, and *** *p* < 0.001, compared with the control group. Distilled water (DW)-administered transgenic (Tg) mice (CON), BJIGT-treated Tg mice (BJIGT), RZ-treated Tg mice (RZ), and combined BJIGT/ RZ-treated Tg mice (BJIGT+RZ). The different number of circles, squares and triangles are indicated the number of sample sot.

As shown in Figure 5A, the number of Nissl-stained motor neurons was significantly increased (by 9-fold) in the ventral horn of the SP in the combination treatment group compared with that in the control group. In addition, compared with the control group, the combination treatment group showed a considerable increase in ChAT-positive neurons by 230% in the SP (Figure 5B). As neuroinflammation leads to the death of motor neurons and induces ALS, we investigated whether the combined BJIGT/RZ treatment could induce an alteration in the neuroinflammation level. The number of Iba-1-positive cells (microglial cells) and GFAP-positive cells (astrocytes) was significantly reduced by 40% and 41%, respectively, in the combination treatment group compared with that in the control group (Figure 5A,B). Moreover, compared with the control group, the combination treatment group showed significantly reduced mRNA levels of the proinflammatory cytokines *IL-1β*, *TNF-α*, and *IL-6* (by 42%, 39%, and 43%, respectively) in the SP (Figure 5C). Interestingly, the *IFN-γ* mRNA level was considerably increased by 120% in the combination treatment group compared with that in the control group. Several studies have demonstrated that TGF-β, which increases in mice and in patients with ALS, is produced by astrocytes to accelerate ALS progression [24]. Here, we found that the expression of TGF-β significantly decreased by 82%, 85%, and 89% in the SP of the BJIGT, RZ, and combination treatment groups, respectively, compared with that in the control group (Figure 5D). These results suggest that the combined BJIGT/RZ treatment exerts neuroprotective effects mediated by inhibiting neuroinflammation in hSOD1^G93A^ transgenic mice.

## 4. Discussion

RZ has been prescribed to patients with ALS for the past two decades; yet its efficacy remains debatable. A previous study suggested that RZ treatment did not significantly improve the lifespan and motor function in three ALS animal models [25]. Thus, based on the findings of previous studies, and considering the multifactorial nature of ALS pathology, we investigated the treatment efficacy of the combination of RZ and BJIGT in an ALS animal model. Our results demonstrated that combination treatment significantly attenuates inflammation, autophagy dysfunction, metabolic dysfunction, and mitochondrial dysfunction in the GC of ALS mice. In addition, this combination treatment has neuroprotective effects mediated through anti-inflammatory activity in the SP of ALS mice.

Inflammation including oxidative stress is recognized as one of the major mechanisms of ALS progression. Turner et al. demonstrated microglial activation in the brains of live patients with ALS using positron emission tomography (PET) [26]. Several reports have suggested that activated glial cells produce proinflammatory cytokines to induce muscle denervation, atrophy, and motor neuron cell death in the end stage of the disease [27]. Furthermore, Xiong et al. demonstrated that oxidative metabolite production is associated with inflammation in ALS [28]. Based on the findings in these previous studies, we investigated inflammation-related markers in the GC and SP of hSOD1^G93A^ mice treated with a combination of RZ and BJIGT (Figure 1 and Figure 5). We found that microglial activation (expression of CD11b) and expression of proinflammatory cytokines (IL-1β and IL-6) were regulated by the combination treatment in the GC of ALS hSOD1^G93A^ mice (Figure 1A). In addition, the combination treatment modulated the activation of microglia and astrocytes, and the production of proinflammatory cytokines (*IL-1β*, *TNF-α*, and *IL-6*) in the SP of hSOD1^G93A^ mice (Figure 5A–C). Furthermore, we found that the expression of TGF-β was significantly decreased in the SP of ALS mice after combination treatment (Figure 5D). Consistently, we found increased *IFN-γ* mRNA levels, which serves as evidence of anti-inflammatory effects. We speculate that the combination treatment inhibits the activation of inflammatory responses by TGF-β and the associated production of *IFN-γ* mRNA. Therefore, we suggest that the combined BJIGT/RZ treatment attenuates inflammatory events and activates antioxidant pathways in the hSOD1^G93A^ mice.

Furthermore, autophagy flux is suppressed in skeletal muscle during the progression of ALS [29]. Rudnick et al. demonstrated that knockout of *ATG7* induced autophagy inhibition and accelerated muscle denervation in SOD1^G93A^ mice [30]. In addition, autophagy dysfunction disrupts the antioxidative stress pathway in the ALS model [31]. Moreover, we found that the levels of the autophagy-related proteins, LC3B and ATG7, were significantly increased in the GC of SOD1^G93A^ mice [32]. However, in this study, we found that the combination treatment with BJIGT and RZ significantly reduced autophagy dysfunction (Figure 1B). These results suggest that combining BJIGT with RZ may lead to increased efficacy in inhibiting inflammation and autophagy dysfunction.

Metabolic homeostasis is also important for maintaining a balance between energy intake and expenditure, and is related to oxidative stress in ALS [33]. Metabolic imbalance has been reported in ALS and contributes to disease progression [34]. PGC-1α is a key regulator of metabolism and mitochondrial biogenesis, and is enriched in the skeletal muscle [35]. Previous studies have demonstrated that the PGC-1α level decreases in ALS and muscle atrophy [36,37]. In addition, Cannavino et al. suggested that the overexpression of PGC-1α in the skeletal muscle prevented metabolic alterations and muscle atrophy [38]. Moreover, exercise attenuated muscle atrophy by inhibiting autophagy dysfunction and regulating the AKT/PGC1-α/FOXO3a pathway in an animal model of muscle atrophy [39]. Consistent with these findings, we found that the combined BJIGT/RZ treatment significantly modulated the expression of PGC-1α, pAKT, and pmTOR in the GC tissue compared with that in the control (Figure 2). These results suggest that the combined BJIGT/RZ treatment regulates metabolic dysfunction by stimulating the antioxidative AKT pathway as the phosphatidylinositol 3-kinase (PI3K/Akt) pathway activates antioxidant gene expression.

The PGC-1α activity is closely associated with mitochondrial dysfunction in skeletal muscles. Muscular mitochondrial dysfunction has been reported to be associated with ALS progression. Furthermore, hSOD1^G93A^ increases oxidative damage and reduces the respiratory activity of mitochondria [40,41]. Notably, mitochondria constantly alter their shape through fusion and fission events to maintain a balance, and an imbalance in these processes causes mitochondrial dysfunction and human diseases. In this study, we found that the expression of the mitochondrial dynamic-related proteins (MFN and DRP1) was significantly upregulated in the Tg group compared with that in the age-matched nTg group. In addition, because the combined BJIGT/RZ treatment regulated metabolic homeostasis in the GC of ALS mice, we assessed whether there were any alterations in mitochondrial function. We found that the expression of mitochondrial fusion- and fission-related protein markers, MFN and DRP1, was significantly decreased in the combined BJIGT/RZ treatment group compared with that in the control group (Figure 3A). Consistently, we demonstrated that the levels of MFN and DRP1 were significantly reduced by the combined BJIGT/RZ treatment in the liver of hSOD1^G93A^ mice [19]. Mitochondria increase or decrease in number by fission or fusion according to the metabolic needs of the cell. Any alterations in these processes deregulate normal mitochondrial functions and bioenergetics, leading to disease onset. Palamiuc et al. reported a significant increase in the ATP level in the asymptomatic stage of ALS in SOD1^G86R^ ALS mice [42]. Our results showed that the ATP level in the GC was restored by the combined BJIGT/RZ treatment (Figure 3B). This finding is consistent with the findings in the liver of combined BJIGT/RZ-treated hSOD1^G93A^ mice in our previous study [19]. ATP production involves glycolysis, tricarboxylic acid cycle, and oxidative phosphorylation. GLUT4 is the major glucose transporter in the skeletal muscle, and its level increases as a result of either exercise or muscle contraction. Our results showed that GLUT4 expression was higher in the Tg group than in the age-matched nTg group. The combination treatment significantly reduced the level of GLUT4 compared with that in the control group (Figure 3C). Glucose-metabolizing pathways, including glycolysis and the TCA cycle, are known to be disrupted in motor neurons and muscles in ALS [42]. Our results showed that the glycogenolysis- and TCA cycle-related enzymes were regulated by the combination treatment (Figure 3E). These results suggest that the combined BJIGT/RZ treatment improves mitochondrial function by regulating mitochondrial dynamics and ATP production in the GC of hSOD1^G93A^ mice.

Skeletal muscle denervation appears in the early stages of ALS, and muscle weakness and atrophy-induced increases in reactive oxygen species (ROS) production are characteristic of ALS [43]; muscle denervation plays a critical role in ALS progression. Previously, we demonstrated that the expression of the muscle denervation-related markers, Prox1 and TGF-β, was significantly increased in the symptomatic stage of ALS (4 months) compared with that in age-matched nTg mice [32]. In this study, we found that the combination treatment significantly reduced the levels of Prox1 and TGF-β compared with those in the control group (Figure 4A). In addition, several studies have demonstrated that increasing *MuSK* activity regulates muscle denervation and the formation of neuromuscular junctions, and improves motor function in ALS mice [44,45]. Based on these findings, we evaluated the mRNA levels of *MuSK*, which protects against denervation; the mRNA level of this protein was significantly increased in the combination treatment group compared with that in the control group (Figure 4B). The neurotransmitter, acetylcholine (secreted by motor neurons), binds to clustered acetyl choline receptors and initiates muscle contraction. Furthermore, *Chrng* is considered a marker of denervation because of the re-expression of the receptor fetal isoform along the length of denervated muscle fibers [46]. In addition, the level of *Myog*, which is a muscle-specific transcription factor, increases during denervation [47]. In this study, we found that the combined BJIGT/RZ treatment attenuated the expression of the muscle denervation markers, *Chrng* and *Myog*, compared with that in the control group in ALS mice (Figure 4B). These results suggest that the combined BJIGT/RZ treatment regulates muscle denervation in the GC of hSOD1^G93A^ mice. The level of CK (a skeletal muscle enzyme) reportedly increases progressively in the serum of animals and patients with early-stage ALS, and this was associated with motor neuron loss, denervation, and muscular atrophy [48,49]. In addition, creatine administration protects motor performance and prolongs survival by antioxidation in hSOD1^G93A^ mice [50]. Interestingly, the combined BJIGT/RZ treatment reduced CK activity and led to an increase in the creatine level in the GC of hSOD1^G93A^ mice (Figure 4C). Therefore, our results show that the combined BJIGT/RZ treatment significantly ameliorates muscle denervation and CK activity by antioxidative effects in the GC of hSOD1^G93A^ mice.

## 5. Conclusions

In summary, the combined BJIGT/RZ treatment attenuated muscle denervation by inhibiting muscle inflammation and regulating metabolic dysfunction in the GC in hSOD1^G93A^ mice. It also protected motor neurons by regulating neuroinflammation in the SP. However, the effects of the combined treatment on the neuromuscular junction (the synaptic connection between motor neuron terminals and skeletal muscle fibers) need to be investigated. In addition, the combined treatment did not appear to affect motor function in this study. In future studies, the administration period should be prolonged to determine whether it leads to changes in motor function and lifespan. Furthermore, the effects of the combined BJIGT/RZ treatment in various ALS patients should be investigated because this study was performed only in hSOD1^G93A^ mice. Statistical analysis should be performed with an appropriate number of samples; it should be based on specific research objectives to avoid illogical interpretations and the dilemma of observing statistically significant effects and the existence of a clinical effect with respect to this particular case as well as when evaluating a large amount of obtained data. Overall, our results provide evidence that BJIGT, used as a dietary supplement in combination with RZ, may have therapeutic effects in patients with ALS.

## Data Availability

Data is contained within the article.
